# Clarification of large‐volume bacterial cultures using a centrifuge‐free protocol

**DOI:** 10.1111/jam.15608

**Published:** 2022-05-16

**Authors:** Toloe Allahghadry, Anders Miki Bojesen, Bradley Joseph Whitehead, Fabio Antenucci

**Affiliations:** ^1^ Department of Veterinary and Animal Sciences, Faculty of Health and Medical Sciences University of Copenhagen Copenhagen Denmark; ^2^ Department of Clinical Medicine – Department of Infectious Diseases Aarhus University Aarhus N Denmark

**Keywords:** bacterial filtration system, centrifuge‐free filtration, filter aid, large‐volume filtration

## Abstract

**Aims:**

To provide a reliable, reproducible and centrifuge‐free filtration protocol for clarification of large volumes of bacterial cultures.

**Methods and Results:**

Four experiments were designed to compare different techniques enabling clarification of *Escherichia coli* cultures using as a benchmark the concentration and quality of bacterial outer membrane vesicles (OMVs). The experiments were designed to examine the performance of different extraction methods on large volume (≥1 L) filtrations of bacterial culture media. Performance parameters included filtration flow rates, sterility testing and characterization of the filtrates by: (i) SDS‐PAGE, (ii) cryogenic transmission electron microscopy, (iii) nanoparticle tracking analysis and (iv) Qubit protein quantification. The experiments revealed that: (i) addition of the filter aid Diatomaceous Earth to the bacterial cultures improved filtration flow rates significantly and eliminated the need for centrifugation prior to filtration; (ii) sterile filtration was successful as no bacterial passage was identified through the membrane filter; (iii) centrifuge‐free filtrates contained an increased amount of OMVs compared to centrifuged filtrates.

**Conclusions:**

In comparison to conventional centrifuge‐based protocols, the clarification method presented has universal applicability for a broad range of microbial extraction procedures, regardless of the volume of culture harvested. Moreover, the decreased amount of OMVs presented in the filtrates following centrifugation step provides an additional argument in favour of a centrifuge‐free approach.

**Significance and Impact of the Study:**

Sterile filtration is a universal method for the clarification of bacterial cultures. Common challenges related to filtration include filter clogging and long processing times, due to limited centrifugation capacity, which can affect product quality. The proposed protocol is likely to ensure a highly effective filtration process and could be a novel approach in improving the filtrate products without the need of centrifugation.

## INTRODUCTION

Extraction of microbial derived products by clarification of bacterial cultures requires a sequence of steps to ensure high yields, high quality and high reproducibility. Generation of crude supernatants has therefore remained a cornerstone of research investigations and industrial processes focusing on microbial derivatives (Lutz et al., 2013; Puertollano et al., 2009; Zhu et al., 2021). Sterile filtration of bacterial cultures has been widely applied in research investigations and commercial applications, for example for the generation of protein‐based pharmaceuticals and vaccines (Cole et al., 2020; Gupta et al., 2020). Two important parameters should be assessed to optimize the process of removing micro‐organisms by filtration; (i) biological parameters such as cell size, concentration and morphology, (ii) technical parameters such as filtration equipment, filter type, pore size, filter material, culture volume, flow rate and cost of the equipment (Bordag et al., 2016). There is thus a considerable and ongoing interest in optimizing the efficiency of sterile filtration, particularly in relation of large volumes relevant for industrial processes, as that may simplify the procedures and bring down production costs. Traditionally, two main methods have been used to remove bacterial cells. One is based on centrifugation relying on cell density, while the other is based on membrane filtration and cell size. Centrifugation utilizes mechanical force to achieve separation and sedimentation of cells and larger particles from the culture medium (Aw et al., 2012; Taulbee & Furst, 2005). Filtration of small volumes of bacterial cultures (<1 L) is normally accomplished by syringe filtration (Walker, 2021). The process can be cumbersome and physically demanding due to filter clogging and is unsuitable for larger volumes (≥1 L) of cultures. To prevent excessive clogging, centrifugation is often employed as a pretreatment to reduce the cell density of the culture. The supernatant obtained is then typically clarified using membrane filtration to achieve optimal results during large‐volume filtration (Benjamin & Lawler, 2013; Doran, 2012; Todaro, 2014). The membranes have a fixed submicrometer pore structure with a uniform size distribution allowing the retention of particles larger than the maximum pore size of the filter (Sarma, 2020). Several types of membrane‐based processes have been developed for research and industrial purposes such as microfiltration (MF), ultrafiltration (UF), nanofiltration (NF), reverse osmosis (RO) and electrodialysis (ED), which rely on separating different solutes based on their size (MF, UF, NF), concentration (RO) or charge (ED), respectively (Batt & Tortorello, 2014). MF is still broadly used all over the world and has been demonstrated to be a reliable and effective method for filtration of small‐volume samples. Besides MF, depth filtration (DF) relies on the same principles for removing particles from liquid (Lacki et al., 2018). DF is based on a combination of fibres (cellulose; polypropylene), diatomaceous earth (DE) as filter aid, and a resin binder used to make flat sheets of filter medium (Lacki et al., 2018). The porous filtration medium used in depth filters retain a large mass of particles throughout the medium before being clogged (Lacki et al., 2018). However, DF has some drawbacks including (i) contamination of filtrate during extended filtration processes due to bacterial growth within the filter matrix, (ii) remaining product within the filter matrix after filtration and iii) media migration during filtration due to possible sloughing off the media fragments (Lacki et al., 2018). Clarification of large‐volume bacterial cultures by MF has considerable drawbacks by being both time‐consuming and labor‐intensive. Large‐volume procedures (≥1 L) are time‐consuming as most high‐speed centrifuges have limited capacity, which means that often the procedure must be repeated (Bordag et al., 2016). Moreover, insufficient documentation of the centrifugation conditions used and the use of different instruments in laboratories makes reproducibility difficult (Zhou, 2012). A critical step in sterile filtration is filter clogging that can occur due to molecular aggregation on the membranes, which poses a risk for deterioration of product quality (van Zwieten et al., 2018). Therefore, there is a clear demand for technologies permitting filtration procedures for large scale of bacterial culture adaptable to a broad array of microbiological workflows to enable high yields, reduced processing times and low production costs.

Bacterial outer membrane vesicles (OMVs) represent bacterial derivatives that can be isolated by clarification of bacterial cultures (Sharma et al., 2008). OMVs are spherical structures containing immune‐active molecules naturally secreted by Gram‐negative bacteria (Schwechheimer & Kuehn, 2015). OMVs have gained significant attention due to their potential in preventing bacterial infections through vaccination (Mancini et al., 2020; Pors et al., 2016). A recent method employed for large‐scale isolation of OMVs from bacterial cultures relies on removal of bacterial cells using sterile filtration, OMV concentration using hydrostatic filtration (HF), and further concentration by Centrifugal Filter Units, which is often required to improve the yield of the OMVs (Antenucci et al., 2020). However, a less labour‐intensive clarification procedure is highly desirable for the production of OMVs. In search of a suitable method, we targeted filtration systems currently employed for clarification of mammalian cell cultures, such as the Sartoclear Dynamics® Lab filtration kit [Sartorius, Goettingen, Germany]. These kits employ vacuum filtration and are primarily designed for the clarification of up to 1 L of mammalian cell cultures (20 × 10^6^ cells/ml) through membrane filters with a pore size of 0.2, 0.22 or 0.45 μm respectively. The membrane of the filter units is composed of polyethersulfones (PES), a material with low protein binding characteristics that can be arranged into an asymmetric matrix, suited for high‐density filtration processes. Pore sizes of 0.22 or 0.45 μm are routinely used for sterile filtration of both eukaryotic and prokaryotic culture media. The Sartoclear Dynamics® Lab filtration kits also include two types of filter aids to prevent low filtration rates due to filter clogging: DE and GMP prefilters. DE, composed of fossilized remains of diatoms, traps particles down to micron levels to increase efficiency in the filtration process (Ghobara et al., 2019). The GMP prefilter is a standard membrane filter made of cellulose acetate with pores of various diameters used to prevent the passage of micro‐organisms (Jeong et al., 2017).

In the current study, we aimed at identifying a reliable, reproducible and centrifuge‐free filtration protocol for the clarification of large‐volume prokaryotic cultures. For this, we successfully tested and optimized an OMV extraction protocol using the Sartoclear Dynamics® Lab system on bacterial cultures up to 1 L. *Escherichia coli* was used as representative of Gram‐negative bacteria due to its wide use in numerous research and industrial processes. For these reasons, we believe the method presented has universal applicability for a broad range of microbial extraction procedures.

## MATERIAL AND METHODS

### Experimental design

Filtration of *bacterial* cultures was performed using Sartoclear Dynamics® Lab filtration kits comprised of a vacuum pump, sterile vacuum filtration units (0.22 and 0.45 μm pore sizes; 21 cm^2^, 62 cm^2^ and 76 cm^2^ filter areas), sterile receiver flasks, buffer flask, multistation stand (Sartolab® MultiStation), sterilized pouches of filter aid (DE) and GMP prefilters. Vacuum was provided by means of negative pressure using a vacuum pump. A series of four sequential experiments were performed to optimize the final protocol (Table 1). The experiments were designed to provide a reliable, reproducible and centrifuge‐free filtration system for the clarification of large volumes of bacterial cultures. In experiments 1, 2 and 3 a multistation stand was employed for hands‐free filtration of upto 6 L of samples simultaneously. Experiments 1 and 2 were pilot experiments and thus performed in small‐scale (50 ml) culture volumes to evaluate feasibility, duration, cost, adverse events and to optimize the filtration process on a small‐scale. Experiments 3 and 4 aimed to scale up the total treatable volume and refine experimental procedures for large‐scale (1 L) purposes. The effect of a centrifugation pretreatment step was examined in experiments 1 and 2. The different approaches were objectively evaluated on the following parameters: (i) comparison of the filtration flow rate; (ii) ability to produce a sterile filtrate; (iii) qualitative protein and OMV content assessed by SDS‐PAGE analysis and cryogenic transmission electron microscopy (cryo‐TEM) imaging, respectively, and (iv) quantification of the protein and OMV content by Qubit protein quantification and nanoparticle tracking analysis (NTA) respectively.

**TABLE 1:** Study design. Filtration of *Escherichia coli* (*E44*Δ) cultures (OD_600_ = 4) using Sartoclear dynamics® lab filtration kits

Experiment no.	Culture volume	Filter unit^a^ pore size (μm)	Centrifugation^b^	Prefiltration treatment	Sample ID
#1	50 ml	0.22	+	None	1–1
				DE (40 g/L)	1–2
				GMP prefilter	1–3
			−	None	1–4
				DE (40 g/L)	1–5
				GMP prefilter	1–6
#2^c^			+	None	2–1; 2–2
	50 ml	0.45	−	DE (20 g/L)	2–3; 2–4
				DE (40 g/L)	2–5; 2–6
#3	1 L	0.22	−	DE (40 g/L)	3–1
				DE (80 g/L)	3–2
#4	1 L	0.22	−	DE (80 g/L)	4–1

Abbreviation: DE, diatomaceous earth.

^a^ Filter unit applied in all experiments was sartolab® RF 50 unit PES (polyethersulfones filter).

^b^ Centrifugation was carried out at 3220 g, 10 min, 4°C.

^c^ Study 2 was performed in duplicate.

### Bacterial cultures

A hypervesiculating *E. coli* (*E44*Δ) mutant strain (ST117 O78:H4) was selected to produce a large quantity of OMVs in the culture medium. The experiments were performed in small‐scale (50 ml) and large‐scale (800 ml) volumes using standard culturing conditions in glass Erlenmeyer flasks (1 L) and a fermenter (7 L; Lambda Minifor) respectively (Todaro, 2014). Briefly, *a single colony of E*. *coli* (*E44*Δ) was subcultured in brain–heart infusion medium (BHI; Gibco) and incubated overnight (ON) under aerobic conditions at 37 °C/160 rpm. On the following day, in the small‐scale experiment, 0.4 ml of the ON culture (0.2% *v/v*) was inoculated in a 1 L flask containing 200 ml preheated BHI broth until reaching OD_600_ = 4 and subsequently divided into 50 ml of the culture volumes. In the large‐scale test, the 7 L flask containing 5 L preheated BHI broth was inoculated with 50 ml of the ON culture (1% *v/v*) to reach OD_600_ = 4. The centrifugation of the selected samples was carried out at 3220 g, 10 min and 4°C. All the samples were filtered using the Sartoclear Dynamics® Lab filtration kits and OMVs were concentrated using HF followed by a further concentration step described in (Antenucci et al., 2020). For an overview of the experimental setup see Table 1.

### Harvesting and filtration

#### Experiment 1

The small‐scale experiments (50 ml samples) using *E*. *coli (E44*Δ) culture were designed to evaluate the filtration efficiency of filter aids (DE and GMP prefilter) on centrifuged and noncentrifuged bacterial cultures. Bacterial cultures were filtered using a membrane pore size of 0.22 μm. The Sartolab® RF 50 unit (0.22 μm, 21 cm^2^, PES filters), multistation stand and a vacuum pump (Microsart e.jet Fluid Pump) were applied in this experiment.

1. Centrifuged group (samples 1–1, 1–2, 1–3): The cultures were divided into 50 ml sample volumes, which were centrifuged (3220 g, 10 min, 4°C) and filtered using: (i) no filter aid, (ii) 40 g/L filter aid (DE) and (iii) GMP prefilter.
2. Noncentrifuged group (samples 1–4, 1–5, 1–6): *The* cultures were divided into 50 ml samples volumes, which were filtered using (i) no filter aid, (ii) 40 g/L filter aid (DE) and (iii) GMP prefilter.

#### Experiment 2

The small‐scale experiments (50 ml samples) of *E*. *coli (E44*Δ) culture were designed to determine the optimal concentration of the selected filter aid (DE) for centrifuge‐free filtration using Sartolab® RF 50 unit (0.45 μm, 21 cm^2^, PES filters), multistation stand and a vacuum pump (Microsart e.jet Fluid Pump). A filter membrane with a pore size of 0.45 μm was used to replicate the syringe filtration method applied previously (Antenucci et al., 2020).

1. Centrifuged group (samples 2–1, 2–2): The cultures were divided into 50 ml sample volumes and centrifuged (3220 g, 10 min, 4°C), in duplicate.
2. Noncentrifuged group (samples 2–3, 2–4; 2–5, 2–6): *The* cultures were divided into 50 ml sample volumes and filtered using 20 and 40 g/L of DE, in duplicate.

#### Experiment 3

A large‐scale experiment of *E*. *coli (E44*Δ) culture (1 L samples; 800 ml of the bacterial culture mixed with DE to reach 1 L) was designed to evaluate and optimize the concentration of DE used in the clarification of bacterial supernatant without a prior centrifugation step. Sartolab® RF 50 unit (0.22 μm, 62 cm^2^, PES filters), a vacuum pump (Microsart e.jet Fluid Pump) and a multistation stand equipped with an adapter were used. The bacterial culture in large‐scale was filtered using a membrane with a pore size of 0.22 μm.

1. Noncentrifuged group (samples 3–1, 3–2): *The* cultures were divided into 800 ml sample volumes, mixed with 40 and 80 g/L of DE to reach 1 L of sample volumes in each and subsequently filtered.

#### Experiment 4

The improvement of the filtration flow rate was evaluated in this experiment. The large‐scale experiment of *E*. *coli* (*E44*Δ) culture (1 L samples; 800 ml of the bacterial culture mixed with DE to reach 1 L) was designed without both a prior centrifugation step and multistation stand, using Sartolab® RF 50 unit (0.22 μm, 76 cm^2^, PES) and a stronger vacuum pump providing sufficient negative pressure (Microsart® maxi.vac).

1. Noncentrifuged group (sample 4–1): 800 ml of the sample volume was mixed with 80 g/L concentration of DE to reach 1 L of sample volume and filtered using a vacuum pump equipped with a buffer flask as a protector, preventing culture flow into the vacuum pump.

### OMV dialysis and concentration

*E. coli* (*E44*Δ) culture crude filtrates produced in this experiment were subjected to OMV isolation as described in (Antenucci et al., 2020). Briefly, the culture crude filtrates were loaded into cellulose ester dialysis membranes with a pore‐size range of 1000 kDa (Religion), and concentrated by HF. The concentrated crude filtrates were then dialysed twice 1:100 (4 h, 4°C) in sterile phosphate‐buffered saline (PBS; Sigma‐Aldrich, St. Louis, MO, USA) and further concentrated by Vivaspin® Turbo 15 ml centrifugal concentrators with a pore‐size range of 100 kDa (PES; Sartorius) used in experiments 2 and 3, and Amicon Ultra‐15 Centrifugal Filter Units with a pore‐size range of 10 kDa (Merck Millipore) used in experiment 4.

### Filtrate characterization

The crude filtrates obtained in all experiments were subsequently harvested, treated by dialysis and subjected to centrifugal concentration, as described previously (Antenucci et al., 2020). The efficiency of the filtration system and the quality of the filtered supernatant obtained were tested for (i) sterility; (ii) SDS‐PAGE, cryo‐TEM, NTA and Qubit protein quantification. Sterility testing of the filtrate was performed in all experiments. OMV characterization tests were carried out in experiments 2, 3 and 4.

#### Sterility testing of the crude filtrate

The test was performed through a spot plating assay (Sanders, 2012). Briefly, the crude filtrates were serially diluted (1:10) in sterile PBS and 10 μl of each dilution was spotted on BHI‐agar plates in triplicate and incubated (37°C/ON). CFU counting was performed the following day.

#### SDS‐page

The isolated OMVs were analysed using 10%–12% SDS‐PAGE gel, previously described in (Antenucci et al., 2020). Briefly, 4× sample buffer and 10× reducing agent were mixed in which 5 μl of OMV samples were added. The reaction mixture was then boiled at 100°C for 10 min and loaded into 10%–12% SDS‐gel wells along with PageRuler™ Plus Prestained Protein Ladder (10–250 kDa; Thermo Fisher). The gel was subjected to electrophoresis at 100 V for 10 min following this at 150 V for 1 h. The protein bands were visualized using Coomassie Blue. Gels were imaged using a ChemiDoc™ XRS+ System and analysed by Image Lab™ Software (Bio‐Rad).

#### Cryo‐TEM imaging

The method applied was performed as previously reported in (Antenucci et al., 2020). Briefly, a hydrophilized lacey carbon 300 mesh copper grid (Ted Pella Inc.) was loaded with 3 μl of OMV solution and blotted using blot force 2, blot and drain times 5.5 and 0 s, temperature 4°C and relative humidity 100% (FEI Vitrobot IV). Subsequently, the sample was mounted into a cryo holder for direct observation at −180°C in a Tecnai G2 20 transmission electron microscope (FEI) at 200 kV. The imaging was conducted using a FEI Eagle camera 4 k × 4 k at variable nominal magnifications.

#### Nanoparticle tracking analysis

The OMV batches were quantified by nanoparticle tracking analysis using NS300 device (Malvern Panalytical) equipped with an sCMOS camera and Blue405 nm laser. The concentration and particle size distribution of the isolated OMVs were detected in accordance with the manufacturer's instructions. Briefly, 1 ml of the diluted sample (1:10,000) in filtered PBS was loaded into a sample chamber via a syringe pump. The camera level of 16 and detection threshold of five were applied to capture the video for 60 s, five times, with the particles per frame of 20–100 at 24.5°C. Data analysis was performed using NTA 3.4 Build 3.4.003 software. The reported results were an average of five 60 s reads.

#### Qubit protein quantification

The isolated OMVs were quantitated by Qubit® 2.0 fluorometer provided with a Qubit™ assay kit containing three standards (Invitrogen) according to the manufacturer's instructions. Briefly, the Qubit reagent was diluted at 1:200 in the Qubit buffer provided in the kit to prepare the Qubit working solution. OMV samples were diluted at 1:5 in the filtered PBS to fall within the calibration curve range. The amount of 10 μl and 1 μl of the standards (each) and the diluted OMV sample were added to 190 and 199 μl of working solution respectively. The assay tubes containing the standard and sample were subsequently vortexed and incubated at room temperature for 15 min. The assay tubes were then inserted into the fluorometer chamber for the data analysis. The curve‐fitting algorithm was used to determine protein concentration based on the relationship between the three standards applied in calibration and the sample dilution factor.

## RESULTS

### Filtration flow rate

**FIGURE 1:**
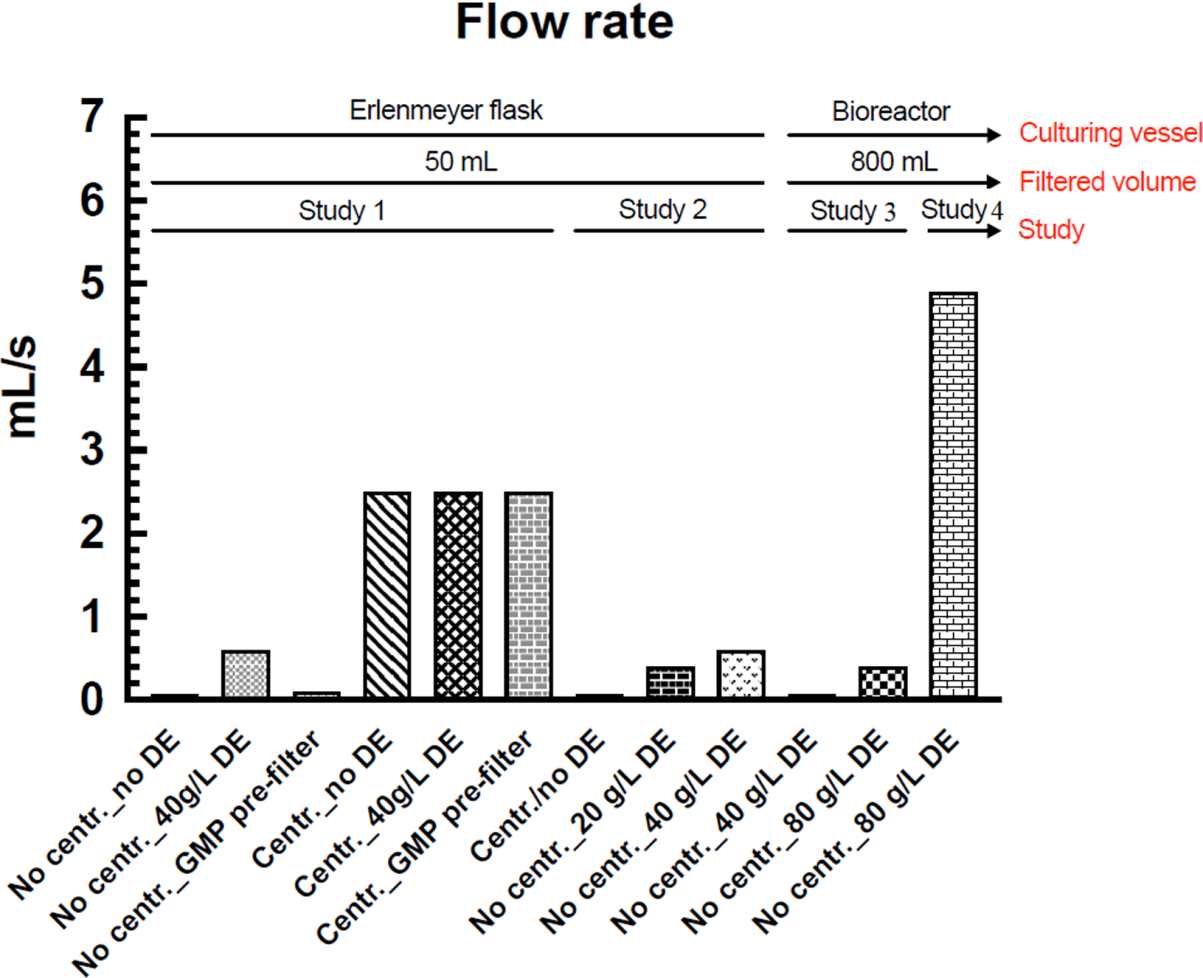
Flow rate (ml/s) in all the studies undertaken. DE, diatomaceous earth; GMP, good manufacturing practices.

The flow rates in experiment 1, 2, 3 and 4 are presented in Table 2 and Figure 1.

**TABLE 2:** Summarized results of the present study in small‐scale and large‐scale production and clarification of bacterial supernatant

Experiment	Centrifugation	Prefiltration treatment [sample ID]	Flow rate (ml/s)	OMV batch	Protein conc. (μg/μl)^a^
#1	+	None [1–1]	2.5	ND	ND
		DE (40 gr/L) [1–2]	2.5		
		GMP prefilter [1–3]	2.5		
	−	None [1–4]	0.07	ND	ND
		DE (40 gr/L) [1–5]	0.6		
		GMP prefilter [1–6]	0.1		
#2	+	None [2–1; 2–2]	0.06	2–1; 2–2	0.82; 0.60
	−	DE (20 gr/L) [2–3; 2–4]	0.4	2–3; 2–4	2.24; 2.23
		DE (40 gr/L) [2–5; 2–6]	0.6	2–5; 2–6	2.47; 2.47
#3^b^	−	DE (40 gr/L) [3–1]	0.07	3–1	2.48
		DE (80 gr/L) [3–2]	0.4	3–2	2.49
#4	−	DE (80 gr/L) [4–1]	5	4–1	3.45

^a^ Protein yields were based on Qubit quantifications.

^b^ Filter aid (DE) was compressed on the surface of the membrane filter (filter cake).

#### Experiment 1

An identical filtration flow rate at 2.5 ml/s was indicated in the centrifuged samples group (50 ml). The filtration flow rates of 0.6, 0.1 and 0.07 ml/s were calculated for the subgroups of supernatant (50 ml) combined with 40 g/L DE, GMP prefilter and without filter aids respectively.

#### Experiment 2

The filtration flow rates of 0.4 and 0.6 ml/s were reported for the filtrate (50 ml) combined with 20 and 40 g/L concentrations of DE respectively. No difference in flow rate (0.6 ml/s) was observed between the membrane filters used in experiment 1 (0.22 μm) and experiment 2 (0.45 μm).

#### Experiment 3

The filtration flow rate of 0.07 and 0.4 ml/s were scored for the filtrate (1 L) combined with DE concentration of 40 and 80 g/L respectively.

#### Experiment 4

The filtration flow rate of 5 ml/s was recorded for the filtrate (1 L) combined with a DE concentration of 80 g/L.

### Sterility testing of the filtrate

No bacterial growth was observed in the filtrates obtained in any of the experiments undertaken.

### Filtrate characterization

**FIGURE 2:**
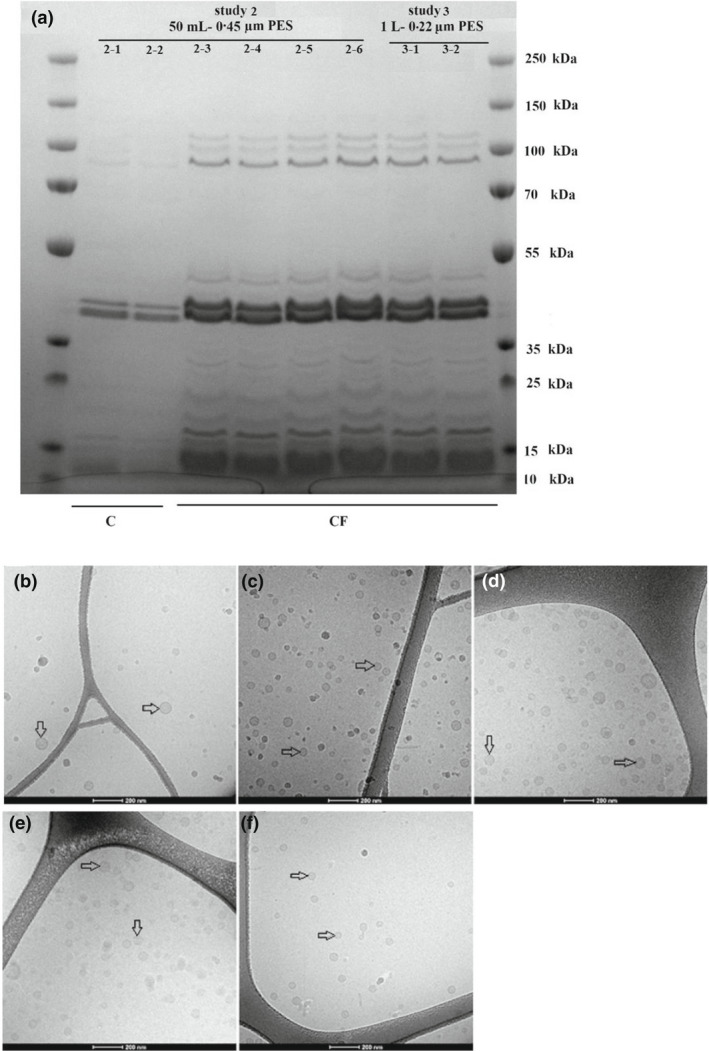
SDS‐PAGE analysis and cryo‐TEM profile of outer membrane vesicles (OMVs) isolated from E. coli (E44Δ) culture in study 2 and 3. (a) SDS‐PAGE analysis of the OMV batches. Lanes legend: 1 and 10: Protein ladder; 2 and 3: OMV batches 2–1 and 2–2 (centrifuged samples without DE as the controls); 4 and 5: OMV batches 2–3 and 2–4 (noncentrifuged samples combined with 20 g/L concentrations of DE); 6 and 7: OMV batches 2–5 and 2–6 (noncentrifuged samples combined with 40 g/L concentrations of DE); 8 and 9: OMV batches 3–1 and 3–2 (noncentrifuged samples combined with 40 g/L and 80 g/L concentrations of DE respectively). (b–f) Cryo‐TEM analysis of OMV batches 2–1 (b), 2–3 (c), 2–5 (d), 3–1 (e) and 3–2 (f). Arrows indicate OMVs. The scale bar represents 200 nm. C, centrifuged sample; CF, centrifuge‐free sample; PES, polyethersulfones filter membrane.

**FIGURE 3:**
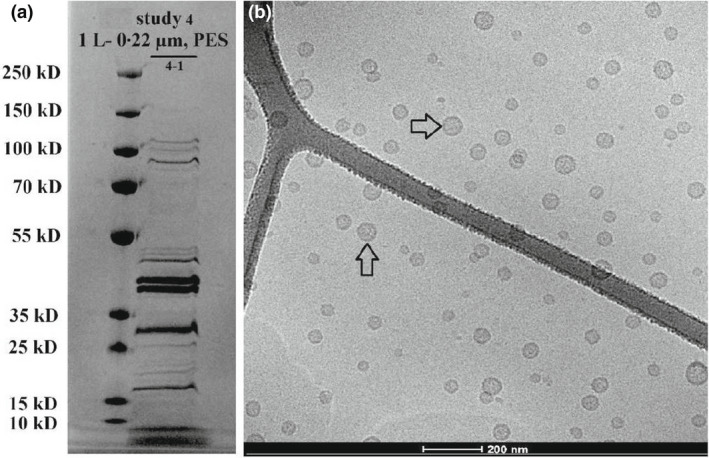
SDS‐PAGE analysis and cryo‐TEM profile of the outer membrane vesicles (OMVs) isolated from *Escherichia coli* (E44Δ) culture in study 4. (a) SDS‐PAGE analysis of the OMV batch. Lanes legend: 1: Protein ladder; 2: OMV batch 4–1 (noncentrifuged sample combined with 80 g/L concentration of DE). (b) Cryo‐TEM analysis of OMV batch 4–1. Arrows indicate OMVs. The scale bar represents 200 nm. PES, polyethersulfones filter membrane.

The SDS‐PAGE analysis of the culture filtrates in experiments 2 and 3 showed that all samples presented similar protein profiles, albeit with different band intensities (Figure 2a). No difference in protein profiles and band intensity was observed between centrifuged samples in experiment 2 and the sample of syringe filtration method performed in a previous study (unpublished data). The results in experiment 2 (Figure 2a) illustrated the increased intensity of the protein bands in the noncentrifuged samples (OMV batches 2–3, 2–4, 2–5, 2–6) when compared with the centrifuged groups (OMV batches 2–1 and 2–2). No visual changes in protein concentrations or banding patterns are detected in Figure 2a between the noncentrifuged samples in experiment 2 (OMV batches 2–3, 2–4, 2–5, 2–6; 50 ml) and experiment 3 (OMV batches 3–1 and 3–2; 1 L). The protein profiles obtained in experiment 4 (OMV batch 4–1; Figure 3a) were similar to experiment 3 performed (OMV batches 3–1 and 3–2; Figure 2a).

**FIGURE 4:**
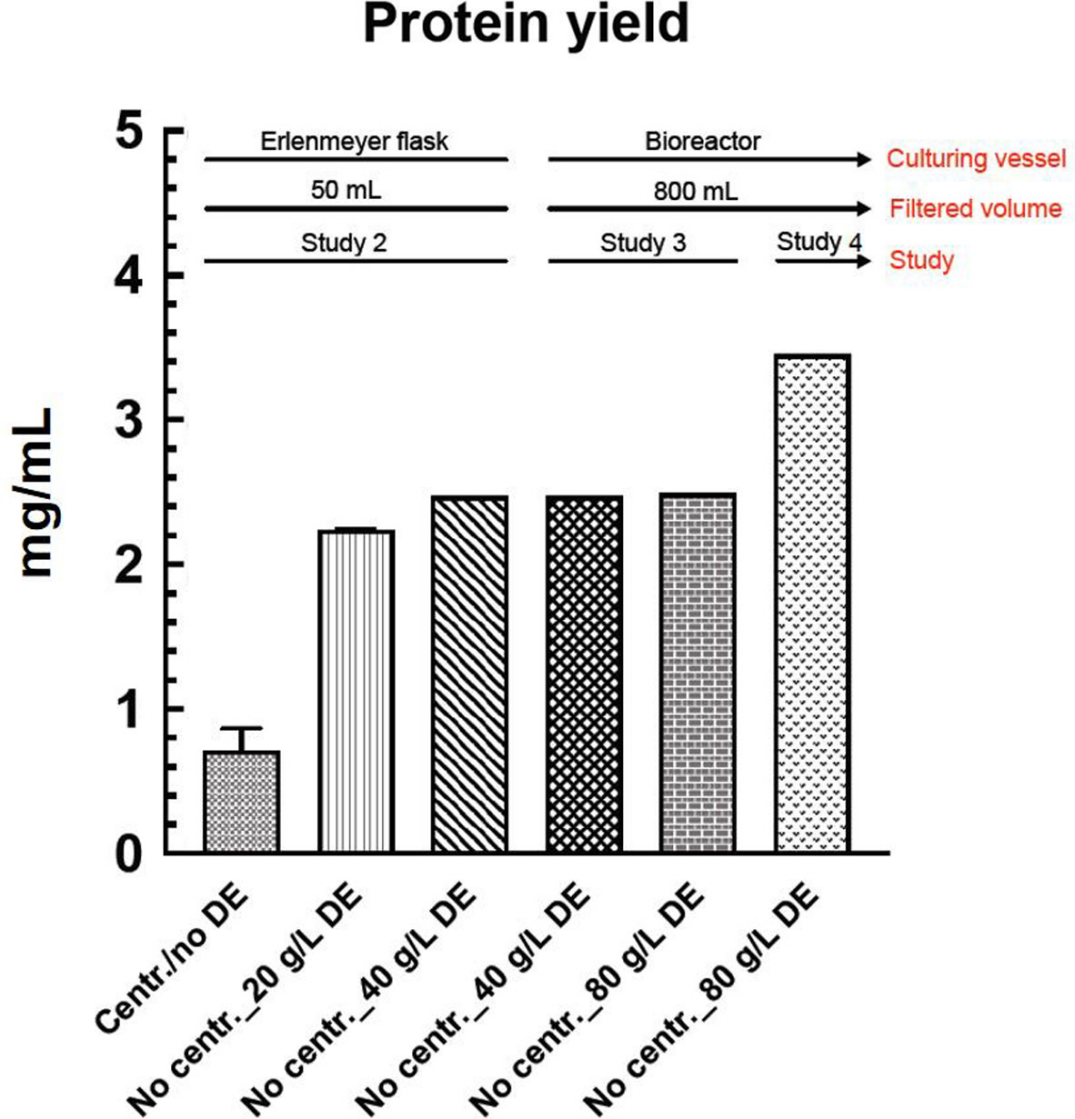
Protein yield obtained in studies 2, 3 and 4 undertaken, measured by qubit protein quantification. DE, diatomaceous earth.

**FIGURE 5:**
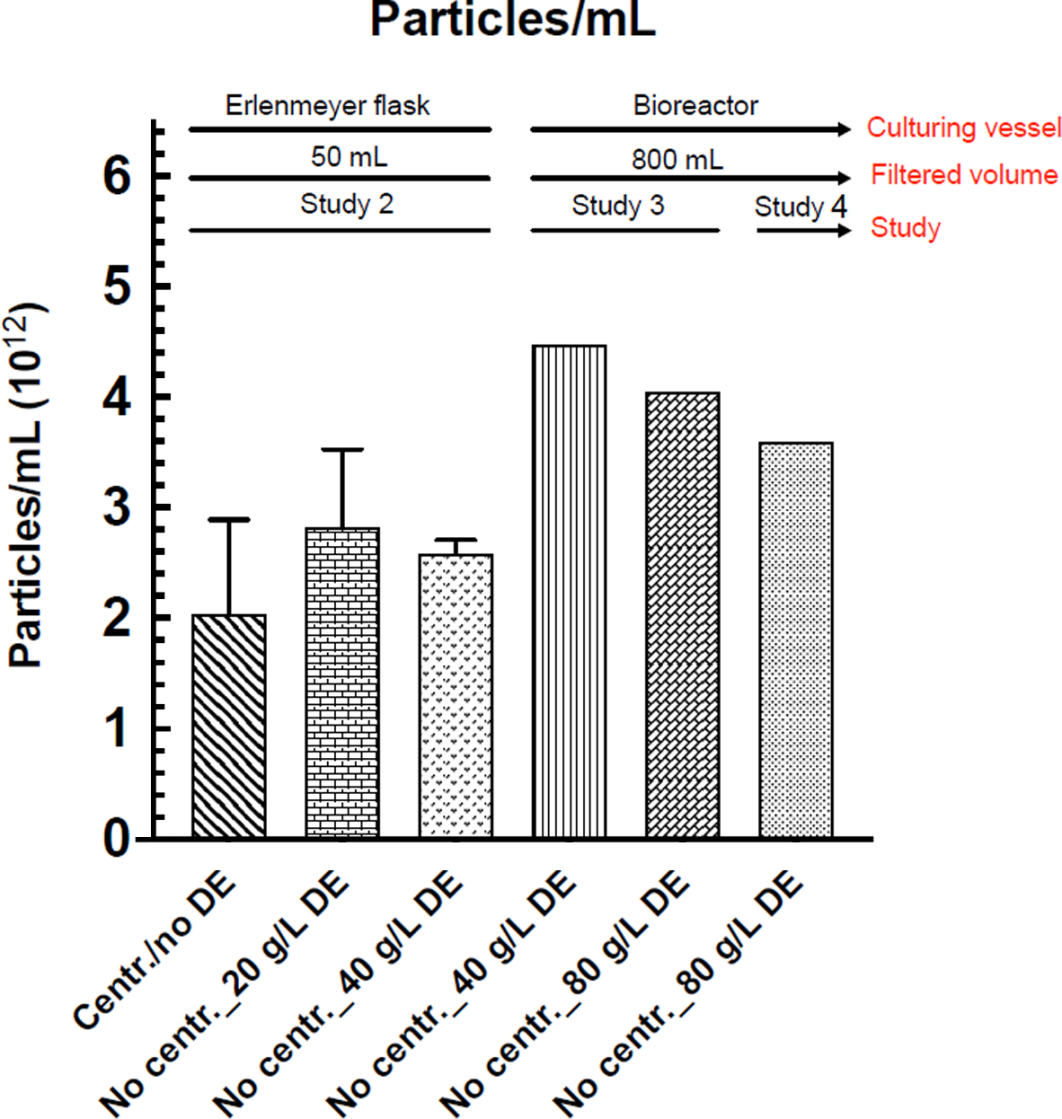
Outer membrane vesicles concentration (particles/ml) in the filtrates isolated from *Escherchia coli* (E44Δ) in study 2, 3 and 4. Measurement by nanoparticle tracking analysis (NTA).

**FIGURE 6:**
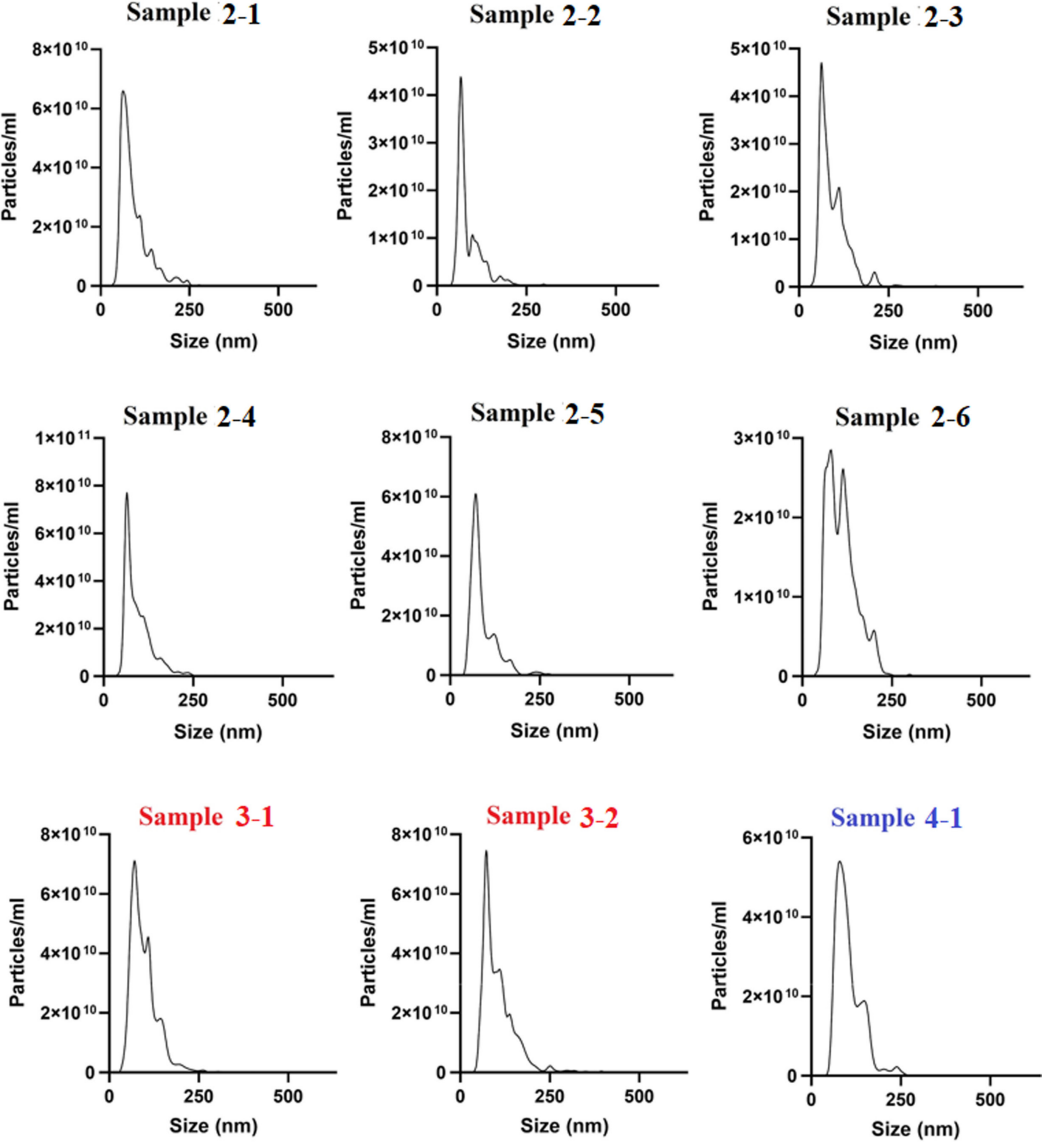
Size and concentration distribution of OMVs obtained from study 2 (2–1, 2–2, 2–3, 2–4, 2–5, 2–6), study 3 (3–1, 3–2) and study 4 (4–1) marked in black, red and blue respectively.

Tha Qubit protein quantification and NTA results in experiments 1, 2, 3 and 4 are presented in Figures 4, 5, 6 and Table 3.

**TABLE 3:** Nanoparticle tracking analysis (NTA) data showing the size and concentration distribution of OMVs isolated in experiment 2, 3 and 4

Experiment no.	OMV batch	Particles diameter (nm)		OMVs concentration (particles/ml)
		Mean	Median	
#2	2–1	95.9 (±1.0)	66.5 (±3.7)	2.64 × 10^12^ (±8.6 × 10^10^)
	2–2	90.4 (±0.9)	68.4 (±2.0)	1.44 × 10^12^ (±1.4 × 10^11^)
	2–3	96.0 (±1.6)	78.7 (±2.4)	2.32 × 10^12^ (±3.2 × 10^11^)
	2–4	96.2 (±1.2)	64.4 (±1.0)	3.32 × 10^12^ (±2.2 × 10^11^)
	2–5	91.3 (±0.8)	71.8 (±2.1)	2.67 × 10^12^ (±1.5 × 10^11^)
	2–6	115.1 (±4.1)	84.1 (±8.4)	2.49 × 10^12^ (±3.9 × 10^11^)
#3	3–1	98.0 (±1.7)	72.5 (±4.6)	4.47 × 10^12^ (±3.6 × 10^11^)
	3–2	107.6 (±1.2)	75.0 (±2.2)	4.04 × 10^12^ (±2.5 × 10^11^)
#4	4–1	105.5 (±1.6)	82.6 (±3.3)	3.59 × 10^12^ (±1.7 × 10^11^)

*Note*: Mean: an average of the particles size measured; Median: the most common particle size measured. Standard error is indicated in the round bracket.

## DISCUSSION

Filtration of bacterial cultures has been extensively studied and optimized, especially in relation to small‐scale filtration (Goreham et al., 2019). We chose to work with *E. coli* as a representative of a laboratory and industrial “workhorse,” as it is used in the production of a very broad range of biological products. However, conventional techniques are not easily scalable for the treatment of large volume bacterial cultures due to the relatively slow flow rate, labour‐intensiveness and being time‐consuming. The Sartoclear Dynamics® Lab kits were originally designed and routinely used for clarifying mammalian cell cultures of upto 1 L. However, their implementation for the filtration of bacterial cultures required optimization of the original protocol. To adapt and integrate this equipment into our system, we designed four experiments aimed at fulfilling the following criteria: (i) parallel and hands‐free filtration of multiple samples simultaneously, (ii) avoidance of centrifugation step using filter aids such as DE or GMP prefilters, (iii) comparably higher flow rates, (iv) an increased time efficiency as compared to the centrifuged‐based methods, (v) minimal contamination risk by employing a complete ready‐to‐use presterilized kit and (vi) minimal impact on yield. The filter aids based on DE and GMP prefilters can increase the flow rate and the efficiency of filtration of bacterial cultures in small volume applications (Jeong et al., 2017; Lagrange et al., 2014). GMP prefilters are a type of porous filter membrane used in removing micro‐organisms, whereas DE is the fused skeletal remains of diatoms with 85% empty space that facilitates liquid flow around the particles and improves the rate of filtration (Lagrange et al., 2014). The efficiency and integrity of a filter are of paramount importance to ensure the quality of filtration. Moreover, the filtration system should ideally allow a high filtration flow rate to minimize the time and labour‐intensiveness (Bruggen, 2018). For these reasons, in this study, we chose both filtration efficiency and flow rate as benchmark parameters to assess the efficacy of the protocol.

Experiment 1 was designed in a small‐scale to evaluate the efficiency of centrifuge‐free filtration of bacterial cultures using filter aids such as DE and GMP prefilters. The flow rates in experiment 1 showed that DE filter aid was more suitable than GMP prefilters for the filtration of bacterial cultures since the latter yielded inferior results (0.1 ml/s) when compared with the application of DE (0.6 ml/s). The optimal concentration of the selected filter aid (DE) for centrifuge‐free filtration of bacterial cultures in the small‐scale and large‐scale were achieved in experiments 2 and 3 respectively. Comparing the flow rates in experiments 2 and 3 indicated 1.5 and 6 times faster flow rates, respectively, when a double amount of DE was applied (Table 2, Figure 1). These results show that the higher DE concentration, the better flow rate which is explained by the highly porous DE acting as a sponge‐like structure and resulting in a more smooth flow rate (Johnson et al., 2020; O'Mahony et al., 2006). However, upon completion of the filtration, a layer composed of DE and bacteria, known as a filter cake, has a tendency to accumulate at the bottom of the filtration unit (Lebleu et al., 2009). The prolonged process of filtration in experiment 3 (1 L in 45 min) using 80 g/L concentration of DE might be due to; (i) the compression of filter cake formed on the surface of the membrane filter, (ii) incomplete vacuum due to the adapters applied and (iii) inadequate mixing of the culture and DE. These important issues that negatively affect flow rate may be addressed by; (i) selecting appropriate filter area (76 cm^2^), (ii) using a vacuum pump capable of generating sufficient negative pressure, (iii) filtration of the samples one at a time without the adapters and (iv) adding DE to the culture followed by thorough mixing. The thicker the cake, the more vacuum is required for filtration, in which mechanical resistance of the filter unit should also be taken into account. To improve filtration flow rate in the filtration of bacterial cultures, the fourth experiment was designed in a large‐scale (1 L, 80 gr/L DE) taking into account the issues encountered in experiment 3. The outcome indicated a fast and trouble‐free filtration flow rate of 5 ml/sec (1 L in ~3.5 min), which is 12.5 times faster than what observed in experiment 3 (1 L, 80 g/L DE). Based on the overall findings, we removed the need for a centrifugation step by employing DE (80 g/L) instead to facilitate the filtration process and prevent filter clogging. The desired vacuum was accomplished without requiring an adapter in the multistation stand. Additionally, the importance of adding DE to the culture and mixing was observed.

Filter units with a pore size of 0.22 μm (experiments 1, 3 and 4) and 0.45 μm (experiment 2) were applied in this investigation. A filtration flow rate of 0.6 ml/s was observed for both filter membranes, 0.22 and 0.45 μm pore sizes, using 40 g/L DE concentration in the small‐scale experiments (sample 1–5 in the experiment 1; samples 2–5 and 2–6 in experiment 2) indicating that pore size did not affect the flow rate per se. The pore sizes were suggested for the filtration of *E. coli* since the overall shape, flexibility and Gram‐type of a bacterium affect its passage through the filter pores (Wang et al., 2008). It was demonstrated that the difference in structure and cell wall composition of bacteria, for example due to the presence of the peptidoglycan layer, may lead to different retention rates during the filtration process (Gaveau et al., 2017). Therefore, Gram‐positive bacteria with a thicker peptidoglycan layer are less deformable potentially increasing the retention rate in comparison to Gram‐negative bacteria (Helling et al., 2020).

Sterility assessment of the filtrates showed no bacterial growth in the crude filtrates obtained by the protocols described in this investigation. This indicates that the filters and procedures employed in this investigation were highly effective in producing cell‐free filtrates regardless of the volume of culture.

The SDS‐PAGE analysis in experiments 2, 3 and 4 showed the same protein profiles albeit with qualitatively different band intensities. It was indicated that the centrifuged samples in experiment 2 (2–1; 2–2) contained a lower protein amount as compared to the noncentrifuged samples obtained in experiment 2 (2–3; 2–4; 2–5; 2–6), experiment 3 (3–1; 3–2) and experiment 4 (4–1) (Figures 2a and 3a), suggesting that the centrifugation step might be responsible for decreasing the amount of OMVs contained in the samples. This observation was also supported by i) the higher protein concentrations in the noncentrifuged samples (more than 2.2 mg/ml) in comparison to centrifuged samples (less than 0.8 mg/ml) as quantified by Qubit protein analysis (Figure 4) and (ii) the higher OMV concentrations in the noncentrifuged samples in comparison to centrifuged samples reported by NTA (Table 3; Figure 5). Nanoparticle tracking analysis is often used to characterize nanoparticles with a size range of 30 ≤ diameters ≤ 600 nm (Maguire et al., 2017). The NTA results showed a size distribution of 60–250 nm for all samples analysed, which are consistent with the size range expected for bacterial OMVs. Furthermore, the OMV size and concentration distributions appeared highly similar and devoid from enlarged OMVs or other artefacts (Figure 6), indicating that the size of OMVs isolated is not influenced by the filtration system employed. The lower limit in the detection of the OMVs using NTA is 60 nm, denoting that this limitation in the assessment of particles below 60 nm must be considered in the NTA results obtained. The results obtained from Qubit protein quantification and NTA are in line with the SDS‐PAGE outcomes, which highlights differences in the product yield between the centrifuged and noncentrifuged samples. This discrepancy could be explained by a loss of OMVs during the centrifugation step, which would represent a clear drawback of performing a centrifugation step prior to filtration. An interesting outcome of this investigation was the observation that despite a larger sample volume (1 L) and smaller filter pore size (0.22 μm), the large sample volume isolation yielded increased amounts of OMVs compared to that observed in the small‐scale experiment (50 ml). Moreover, the qualitative analysis of the OMVs isolated from the developed system indicated no large OMVs (diameter ≥ 150 nm), OMV aggregates or artefacts in cryo‐TEM profiles (Figure 2b–f and 3b), which was consistent with the NTA results. The overall findings indicated that the Sartoclear Dynamics® Lab filtration kit (0.22 μm filter pore size; 76 cm^2^ filter area; vacuum pump providing sufficient negative pressure [>750 mbar] and 80 g/L concentration of DE) could clarify at least 1 L of bacterial culture at a flow rate of 5 ml/s without prior centrifugation. Based on the results achieved, the proposed protocol is likely to ensure a highly effective filtration process regardless of the volume of culture treated. Hence, the optimized filtration system introduced can play an important role in enhancing the efficiency of the entire clarification process.

In conclusion, our investigation clearly indicated the efficacy of a single‐step pipeline for the clarification of large‐volume bacterial cultures using a modified protocol of the Sartoclear Dynamics® Lab kits. The filtration technique allows for more efficient clarification of large volumes of bacterial cultures as compared to conventional centrifuge‐based protocols. Our data also suggest that centrifugation might lead to a decrease in the amount of OMVs present in the filtrates, thus providing an additional argument in favour of a centrifuge‐free approach.

## CONFLICT OF INTEREST

The authors declare no conflict of interest.
